# The influence of different bariatric surgeries on male sex hormones and semen parameters among infertile obese male patients: an observational study

**DOI:** 10.1186/s12893-026-03710-9

**Published:** 2026-05-23

**Authors:** Mahmoud Azhary, Mohamed Hassan Ali, Mohamed Ahmed AbdELsalam, Mohamed Elshal, Ahmed Maher Abdelmonim, Ehab Fathy Ahmed

**Affiliations:** 1 General and Laparoscopic Surgery, Cairo University Hospital, Kasr Al-Ainy, Cairo, 11562 Egypt; 2 Department of Andrology, Sexology and STDs, Faculty of medicine - Cairo University Cairo, Cairo, 11562 Egypt

**Keywords:** Obesity, Male infertility, Metabolic and bariatric surgery, Sleeve gastrectomy, One-anastomosis gastric bypass, Roux-en-Y gastric bypass

## Abstract

**Background:**

This research examines the impact of bariatric procedures on male sex hormones and semen parameters in infertile men with obesity. Obesity adversely affects male fertility by causing hormonal imbalances and worsening semen quality. Metabolic and bariatric surgery (MBS) offers sustained weight loss and potential reversal of these abnormalities.

**Methods:**

This prospective case series included 43 infertile men with severe obesity who underwent sleeve gastrectomy, One-anastomosis gastric bypass (OAGB), or Roux-en-Y gastric bypass. All participants had a BMI ≥ 35 kg/m² and a history of infertility for over one year. Semen analysis and hormonal profiling (FSH, LH, total testosterone, estradiol [E2], and prolactin) were conducted preoperatively and at 3, 6, and 12 months postoperatively.

**Results:**

Significant weight loss was observed at all follow-up points (*p* ≤ 0.003). Improvements were noted in semen motility, progressive motility, vitality, and abnormal forms (all *p* ≤ 0.003). Serum testosterone levels increased, while estradiol levels decreased significantly (*p* ≤ 0.003). Changes in FSH, LH, and prolactin were statistically insignificant.

**Conclusions:**

Metabolic and bariatric surgery (MBS) is associated with marked improvements in semen quality and serum testosterone levels, supporting its role as an effective therapeutic strategy for obesity-related male infertility. No pregnancies were recorded during the 12-month follow-up.

**Trial registration:**

Not applicable.

## Introduction

Obesity affects more than 650 million adults worldwide and is a major modifiable risk factor for male infertility [1, 2]. Excess adiposity disrupts the hypothalamic–pituitary–testicular axis, increases aromatization of testosterone to estradiol, and promotes systemic inflammation and oxidative stress—all of which impair spermatogenesis [2–9]. Although large cohort studies indicate a dose-dependent association between increasing body mass index (BMI) and altered sperm DNA integrity, evidence regarding conventional semen parameters remains inconsistent [3, 4, 10, 11]. The ‘parameters’ assessed in this study include semen motility, vitality, morphology, and concentration.

Lifestyle interventions may achieve modest weight reduction and partial hormonal improvement, but their effects on male reproductive outcomes are limited [12–14]. In contrast, metabolic and bariatric surgery (MBS) provides sustained weight loss and significant metabolic improvements, making it a promising therapeutic option for obesity-related hypogonadism and subfertility [5, 15–17].

Previous studies have shown postoperative increases in testosterone and reductions in estradiol; however, findings regarding semen quality remain variable, and deterioration has been reported in some cases [9, 11, 18–23].

Multiple bariatric procedures are currently used, each producing weight loss through distinct mechanisms. Sleeve gastrectomy (SG) is primarily restrictive, whereas one-anastomosis gastric bypass (OAGB) and Roux-en-Y gastric bypass (RYGB) combine restriction with varying degrees of mal-absorption and metabolic modulation [15, 16]. Given these physiological differences, their effects on reproductive hormones and semen quality may not be uniform. Yet, comparative data evaluating these procedures specifically in infertile men remain scarce [9, 11, 18, 21].

This study prospectively assesses the impact of SG, OAGB, and RYGB on serum sex hormones and semen parameters in infertile men with obesity. While different bariatric procedures were included, the primary analytical focus was the prospective within-subject assessment of reproductive outcomes, with procedure-specific comparisons explored as secondary analyses.

## Methodology

### Study design and setting

This study was designed as a prospective observational case series without a non-surgical control group because all eligible patients had already been scheduled for metabolic and bariatric surgery based on established clinical indications. We did not include a non-surgical comparator group because the patients who came to our bariatric clinic were already scheduled for metabolic and bariatric surgery based on clinical indications, symptom severity, and patient preference. Therefore, it would not have been ethical or clinically appropriate to withhold surgery or assign patients to non-surgical management. Consequently, the study design mirrors actual clinical practice, and the results must be understood within the parameters of this intrinsic selection framework.

This was a single-center, prospective observational case series conducted at a tertiary university hospital between January 2021 and February 2025. A total of 43 infertile men with obesity were included. Participants underwent metabolic and bariatric surgery (MBS) based on clinical indications and surgeon preference; therefore, randomization was not feasible. To reduce potential confounding across procedures—sleeve gastrectomy (SG), one-anastomosis gastric bypass (OAGB), and Roux-en-Y gastric bypass (RYGB)—patients were matched post hoc for age (± 3 years), baseline body mass index (BMI; ±2 kg/m²), and infertility type (primary vs. secondary).

Comparisons between surgical procedures were not the primary objective of the study and were conducted as exploratory analyses due to limited subgroup sample sizes.

The primary outcomes were changes in semen parameters (motility, vitality, concentration, morphology) and serum sex hormones (total testosterone, follicle-stimulating hormone [FSH], luteinizing hormone [LH], estradiol [E2], and prolactin) at 3, 6, and 12 months postoperatively. Secondary outcomes included BMI and metabolic markers. The study adhered to STROCSS 2021 reporting guidelines [18].

This study was not registered in a clinical trial registry, as it was an observational case series without an interventional protocol.

### Participant characteristics

**Fig. 1:**
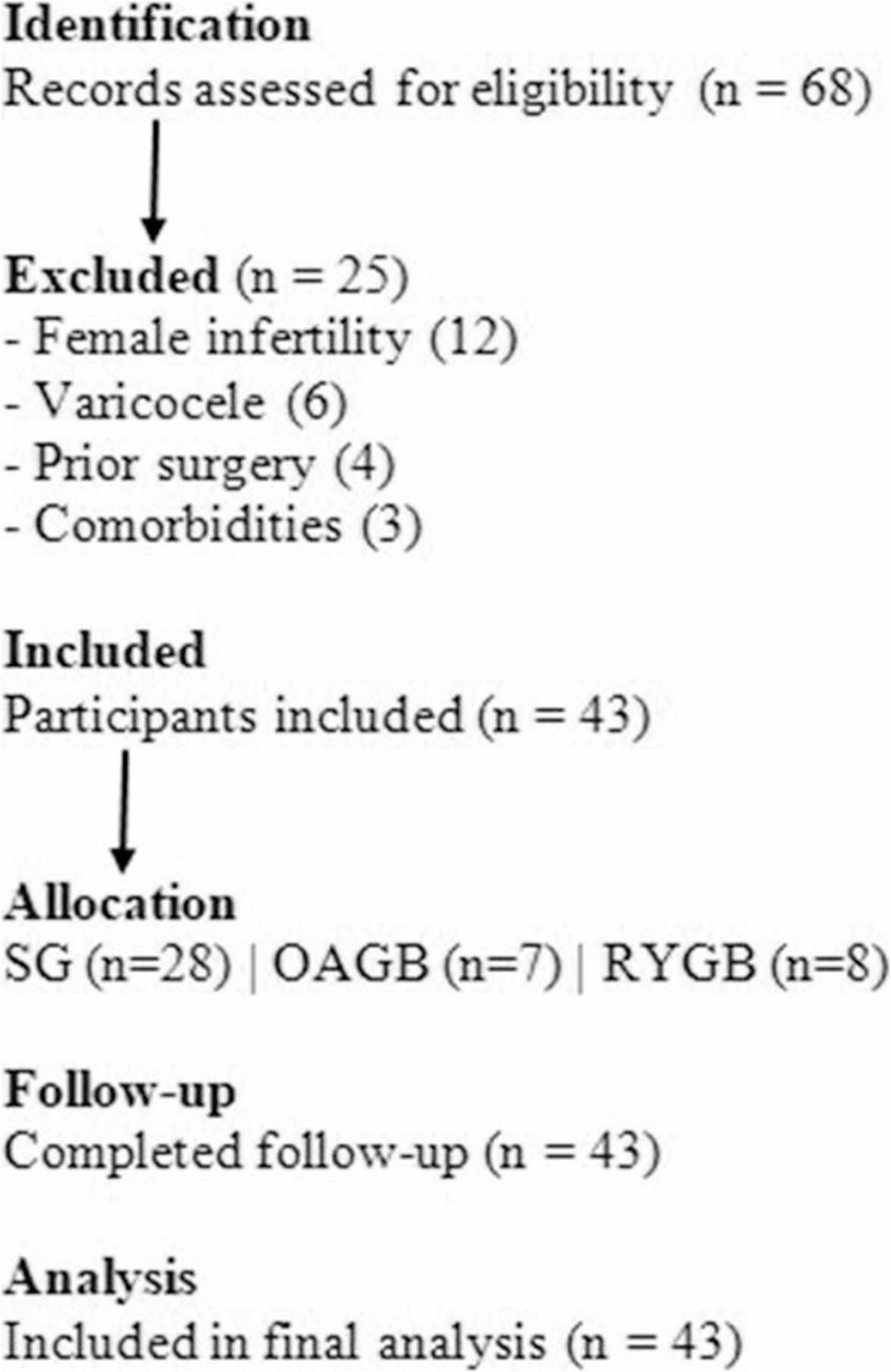
Flowchart of participant recruitment, exclusions, allocation, and follow-up

Among 68 screened men, 43 met eligibility criteria and consented to participate (recruitment rate: 63.2%) (Fig. 1).

Inclusion criteria included men aged 18–65 years, body mass index (BMI) ≥ 35 kg/m², primary or secondary infertility lasting ≥ 1 year, surgical fitness, and willingness to comply with long-term follow-up.

Exclusion criteria were: Prior abdominal or pelvic surgery, history of radiotherapy or chemotherapy, Leukocytospermia, significant uncontrolled comorbidities, heavy smoking, clinical or subclinical varicocele, Testicular atrophy (< 12 mL), structural genital abnormalities, psychological or sexual dysfunction, sleep disorders, female-factor infertility.

Varicocele was defined clinically and confirmed by Doppler ultrasound [19]. Reasons for exclusion included female-factor infertility (*n* = 12), varicocele (*n* = 6), prior surgery (*n* = 4), and uncontrolled comorbidities (*n* = 3).

No participants were lost to follow-up, and no missing data were recorded throughout the study.

### Intervention

#### Preoperative evaluation

All participants underwent standardized preoperative assessment, including medical history, demographic data, comorbidities, and laboratory investigations. Written informed consent was obtained prior to participation.

#### Laboratory and imaging investigations

Baseline testing included complete blood count, fasting blood sugar, HbA1c, liver and renal profiles, and coagulation studies. Additional evaluations included abdominal ultrasound, chest radiography, and electrocardiography.

#### Semen analysis

Semen samples were collected following 2–7 days of abstinence. Two samples per patient were obtained and averaged. Analyses were performed according to the WHO 6th edition manual [20]. Parameters assessed included: total motility, progressive motility, morphology (Tygerberg criteria; normal ≥ 4%), vitality (eosin–nigrosin staining).

All assessments were performed by a single blinded embryologist to minimize inter-observer variability.

#### Hormonal profiling

Serum FSH, LH, total testosterone, prolactin, and estradiol were measured using chemiluminescent immunoassay (Cobas e601, Roche Diagnostics). Inter-assay coefficient of variation was < 8%. Sex hormone-binding globulin (SHBG) was measured, and free testosterone was calculated using the Vermeulen formula.

Free testosterone was calculated using the Vermeulen formula to support internal assessment of androgen status; however, due to strong collinearity with total testosterone and to avoid redundancy in outcome reporting, free testosterone was not analyzed as a separate primary endpoint.

#### Scrotal ultrasound

Ultrasonography was used to assess testicular volume and exclude pathological abnormalities. Only participants with normal bilateral testicular architecture were included.

- *Surgical Procedures*: All surgeries were performed laparoscopically under general anesthesia.
- *SG*: 36 Fr bougie; resection beginning 4 cm proximal to pylorus
- *OAGB*: 200 cm biliopancreatic limb, 300 cm common channel
- *RYGB*: 75 cm biliopancreatic limb, 100 cm alimentary limb

#### Postoperative care

Patients received early mobilization, proton pump inhibitors for 6–8 weeks, and staged dietary advancement (liquid → soft → regular diet). Standard multivitamin supplementation included Centrum Silver^®^, vitamin D, calcium carbonate, B-complex vitamins, and whey protein. Antioxidants and fertility supplements were prohibited unless medically indicated.

#### Follow-up schedule

Participants were evaluated at 3, 6, and 12 months postoperatively. Each visit included:

#### Physical examination

Semen analysis, serum sex hormone testing and BMI were recorded at each visit.

### Statistical analysis

Changes in semen parameters and hormonal measurements over time were assessed using linear mixed-effects models with fixed effects for time, age, baseline BMI, and surgical procedure, and random intercepts for individual participants. Bonferroni correction was applied to adjust for multiple comparisons. Effect sizes and 95% confidence intervals were reported. Sensitivity analyses excluded three individuals with transient semen deterioration.

All statistical analyses were performed using two-sided hypothesis testing, with a significance level of α = 0.05.

Data analysis was conducted using SPSS version 28 (IBM Corp.) and R version 4.3.1.

No a priori sample size calculation was performed due to the exploratory design. With 43 participants and an anticipated effect size of Cohen’s d ≥ 0.6, statistical power exceeded 80% for detecting changes in testosterone levels at α = 0.05.

### Ethical considerations

Ethical approval was obtained from the faculty of medicine, Cairo University Research Ethics Committee (Approval No. MD-296-2021). All participants provided written informed consent prior to enrollment. Data were collected, stored, and analyzed confidentially in accordance with the Declaration of Helsinki.

This study did not require clinical trial registration as it was an observational case series with no interventional allocation.

## Results

Our study included 43 infertile men with obesity. The majority (65.1%) had primary infertility, while the remaining 34.9% had secondary infertility. Regarding surgical procedures, most participants underwent sleeve gastrectomy (SG; 65.1%), followed by Roux-en-Y gastric bypass (RYGB; 18.6%) and one-anastomosis gastric bypass (OAGB; 16.3%) (Table 1; Fig. 1).

**Table 1:** Type of infertility and type of operation

		Count	%
Type of infertility	Primary	28	65.1%
	Secondary	15	34.9%
Type of operation	Sleeve gastrectomy	28	65.1%
	Mini-bypass	7	16.3%
	Classic-Bypass	8	18.6%

Data presented as numbers and percentages as appropriate

### Changes in body size and composition

BMI decreased significantly from baseline to 12 months (59.9 to 30.4 kg/m²; *p* < 0.001). Most participants transitioned from severe obesity to moderate obesity or overweight categories (Table 2). This reduction shifted most participants from severe obesity to lower BMI categories.

**Table 2:** Shows effect of operation on BMI over 3,6 and 12 months

	Mean	SD	Median	Minimum	Maximum	P value compared to before
BMI Before	59.89	11.25	60.20	40.80	77.50	----
BMI After (3 months)	49.16	9.62	50.01	34.66	73.29	< 0.001
BMI After (6 months)	39.10	7.22	37.70	28.20	53.00	< 0.001
BMI After (12 months)	30.44	4.33	30.20	23.50	39.80	< 0.001

*Abbreviation BMI* Body Mass Index

Data presented as mean ± SD

*P* values were derived from the linear mixed-effects models

### Semen parameters

**Fig. 2:**
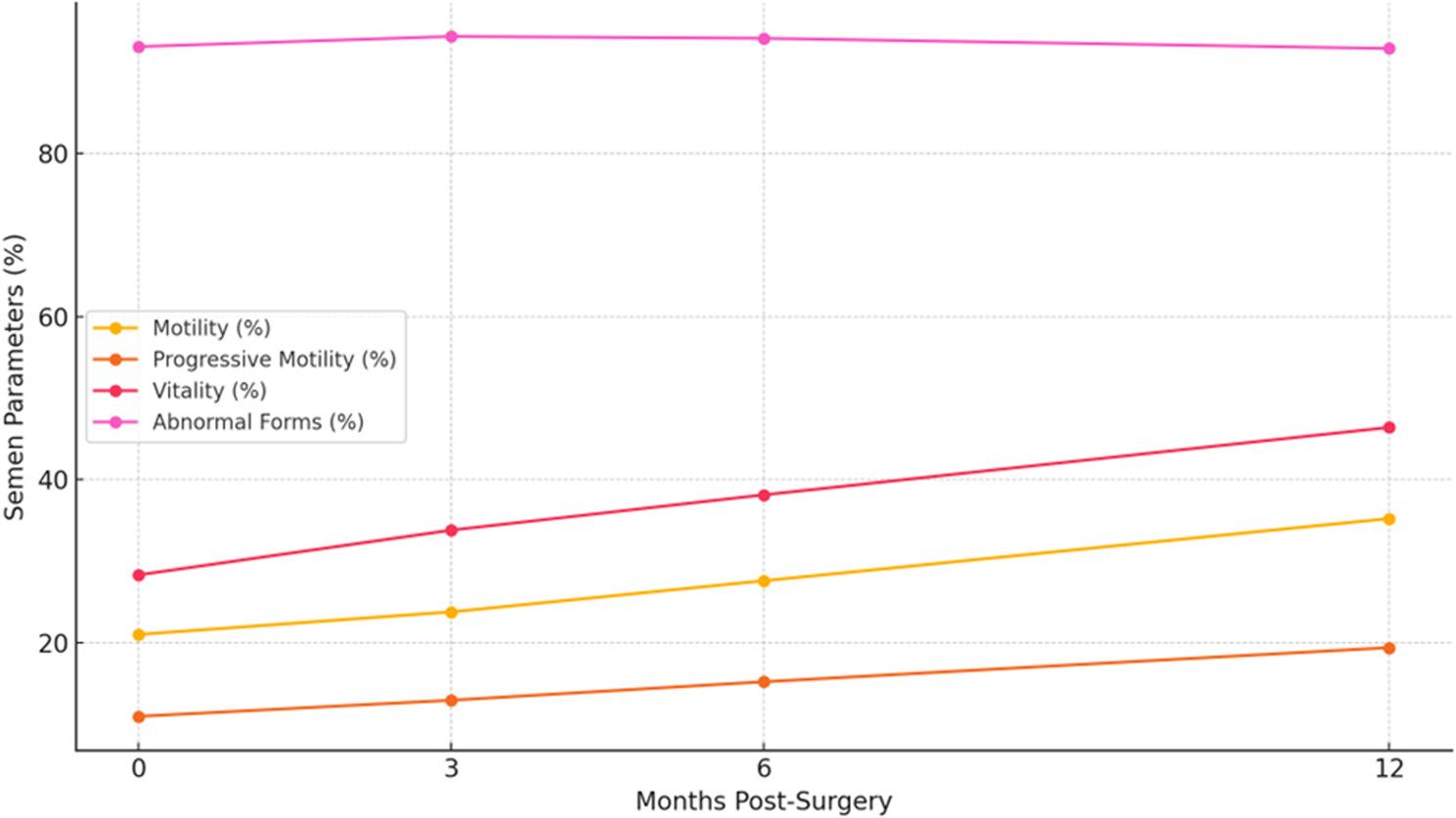
Changes in semen parameters (total motility, progressive motility, vitality, and abnormal forms) over 12 months post-surgery

Significant improvements were observed in total motility, progressive motility, vitality, and abnormal forms at all postoperative assessments (*p* < 0.001), the high percentage of abnormal forms (93–94%) reflects the percentage of morphologically abnormal sperm, and that the statistically significant p-values reflect redistribution/variance reduction rather than a large mean change. Sperm concentration did not change significantly (*p* = 0.991). Three participants experienced transient reductions in semen quality between 6 and 9 months; micronutrient deficiencies (zinc or selenium) were identified and corrected. (Table 3; Fig. 2).

**Table 3:** Shows effect of operation on SEMEN ANALYSIS over 3,6 and 12 months

	Mean	SD	Median	Minimum	Maximum	*P* value compared to before
Effect of operation on SEMEN ANALYSIS (Concentration)						
Conc. Before	22.09	26.03	10.75	0.00	89.80	----
Conc. After (3 months)	21.08	24.35	10.16	0.00	85.92	0.838
Conc. After (6 months)	20.81	24.05	10.71	0.00	87.87	0.766
Conc. After (12 months)	21.00	24.93	11.12	0.00	89.54	0.991
Effect of operation on SEMEN ANALYSIS (Motility)						
Motility Before	21.02	13.20	20.00	0.00	54.00	----
Motility After (3 months)	23.78	14.76	19.68	0.00	59.37	0.003
Motility After (6 months)	27.60	17.06	26.00	0.00	68.00	0.001
Motility After (12 months)	35.22	22.51	37.00	0.00	78.00	< 0.001
Effect of operation on SEMEN ANALYSIS (PR)						
PR Before	10.98	8.17	10.00	0.00	31.00	----
PR After (3 months)	12.95	8.59	10.26	0.00	36.53	< 0.001
PR After (6 months)	15.22	9.85	13.00	0.00	39.00	< 0.001
PR After (12 months)	19.40	12.87	16.00	0.00	54.00	< 0.001
Effect of operation on SEMEN ANALYSIS (Abnormal form)						
Abn. Forms Before	93.07	20.37	98.00	0.00	100.00	----
Abn. Forms After (3 months)	94.32	14.68	96.87	0.00	99.31	0.001
Abn. Forms After (6 months)	94.09	14.46	96.00	0.00	100.00	< 0.001
Abn.Forms After (12 months)	92.84	14.42	95.00	0.00	100.00	< 0.001
Effect of operation on SEMEN ANALYSIS (Vitality)						
Vitality Before	28.32	15.84	32.00	0.00	54.00	----
Vitality After (3 months)	33.80	15.06	37.19	0.00	58.94	< 0.001
Vitality After (6 months)	38.13	15.31	42.00	0.00	60.00	< 0.001
Vitality After (12 months)	46.40	18.22	49.00	0.00	81.00	< 0.001

Data presented as mean ± SD

*P* values were derived from the linear mixed-effects models

The high percentage of abnormal forms (93–94%) reflects.

### Hormonal profile

**Fig. 3:**
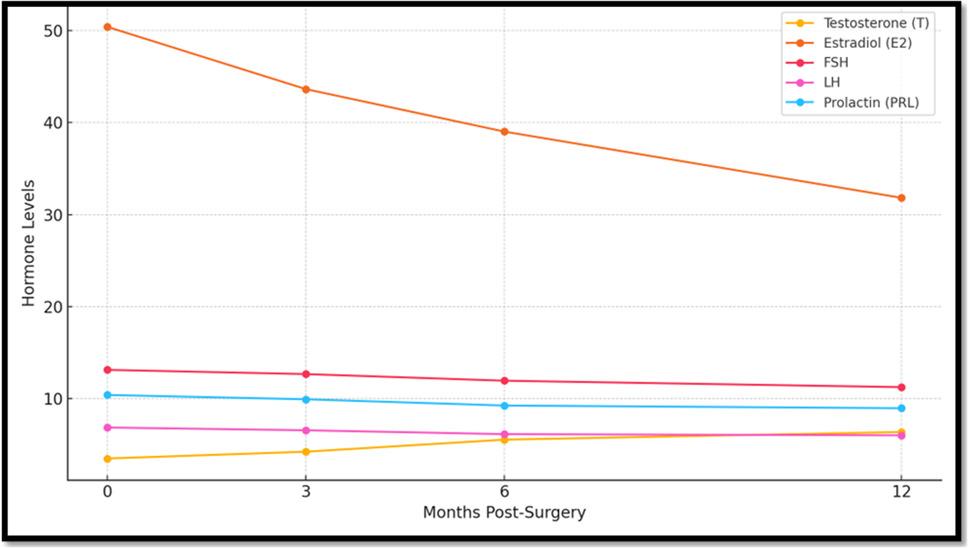
Changes in serum testosterone and estradiol levels before and at 3, 6, and 12 months after bariatric surgery

Testosterone increased significantly (mean + 38.7%; *p* < 0.001), while estradiol decreased (− 33.5%; *p* < 0.001). FSH, LH, and prolactin did not show significant changes (*p* > 0.05). (Table 4; Fig. 3). 

**Table 4:** Shows effect of operation on SEX HORMONES over 3, 6 and 12 months

	Mean	SD	Median	Minimum	Maximum	*P *value compared to before
Effect of operation on FSH HORMONE						
FSH (mIU/mL) Before	13.11	4.29	12.12	6.32	21.44	----
FSH (mIU/mL) After (3 months)	12.65	4.26	11.96	6.34	20.91	0.131
FSH (mIU/mL) After (6 months)	11.93	5.03	10.46	4.93	20.55	0.067
FSH (miU/mL) After (12months)	11.23	6.98	8.98	2.78	26.51	0.064
Effect of operation on LH HORMONE						
LH (mIU/mL) Before	9.63	4.50	9.24	2.29	17.04	----
LH (mIU/mL) After (3 months)	9.68	3.72	10.16	2.54	16.84	0.917
LH (mIU/mL) After (6months)	10.25	4.17	10.88	2.77	16.89	0.406
LH (miU/Ml) After (12 months)	10.96	6.90	8.90	1.98	22.10	0.314
Effect of operation on T HORMONE						
T (ng/mL) Before	4.65	1.77	4.55	1.83	7.87	----
T (ng/mL) After (3 months)	5.65	1.72	5.57	2.24	8.71	< 0.001
T (ng/mL) After (6 months)	6.15	1.89	6.53	2.63	9.17	< 0.001
T (ng/mL) After (12 months)	7.12	2.62	8.12	2.10	9.99	< 0.001
Effect of operation on PRL HORMONE						
PRL (ng/mL) Before	9.97	4.71	9.60	2.16	17.75	----
PRL (ng/mL) After (3 months)	10.01	4.12	9.88	2.98	17.25	0.949
PRL (ng/mL) After (6 months)	9.76	4.46	9.58	2.17	17.93	0.833
PRL (ng/mL) After (12 months)	10.20	6.72	9.97	2.17	19.64	0.873
Effect of operation on E2 HORMONE						
E2 (pg/mL) Before	40.60	11.38	40.77	14.50	59.84	----
E2 (pg/mL) After (3 months)	31.57	11.49	33.27	12.66	59.09	< 0.001
E2 (pg/mL)After (6 months)	25.01	9.88	25.14	10.72	44.37	< 0.001
E2 (pg/mL) After (12 months)	16.05	10.29	11.74	4.56	43.50	< 0.001

*Abbreviations FSH*Follicular Stimulating Hormone, * LH *Luteinizing Hormone,  *T* Teststeron Hormone,  *PRL* Prolactin, *E2* Estrogen hormone

Data presented as mean ± SD

*P* values were derived from the linear mixed-effects models

Clinical Implications—and although no pregnancies occurred during the 12-month follow-up, and no couples pursued fertility treatments, the physiological improvements we documented are promising. The combination of better sperm motility, vitality, and morphology—alongside normalized testosterone-to-estradiol ratios—suggests that metabolic and bariatric surgery may help reset the biological foundations of male fertility in men with obesity. That said, without data on actual conception or live birth rates, we can’t yet link these changes directly to improved fertility outcomes. These findings suggest that MBS may contribute to improvements in reproductive biology beyond weight reduction alone, primarily by restoring sperm function and correcting the hormonal imbalance between testosterone and estradiol (Table 5).

**Table 5:** Comparison of Hormonal and Semen Parameter Changes across Surgical Groups at 12 Month

Parameter	SG (*N* = 28)	OAGB (*N* = 7)	RYGB (*N* = 8)	*P* value
BMI (Δ)	-28.5	-31.2	-30.8	0.12
TT (Δ, ng/mL)	+ 1.8 ng/mL	+ 2.1 ng/mL	+ 2.05 ng/mL	0.08
E2 (Δ, pg/mL)	-15 pg/mL	-22 pg/mL	-20 pg/mL	0.03*
Motility (Δ, %)	+ 18%	+ 24%	+ 22%	0.04*

*Abbreviations TT *Total Testosterone, *E2*Estradiol

Data presented as mean ± SD

*P* values were derived from the linear mixed-effects models

Exploratory subgroup analysis; study not powered for between-procedure comparisons

#### Limitations

These between-procedure comparisons should be interpreted with caution, as subgroup sample sizes were small and the analyses were exploratory in nature.

## Discussion

Comparisons between surgical procedures should be interpreted with caution, as these analyses were exploratory and the study was not powered for definitive between-procedure comparisons.

This study demonstrates that metabolic and bariatric surgery (MBS) leads to measurable improvements in key reproductive parameters among infertile men with obesity. Total and progressive sperm motility, vitality, and morphology improved over 12 months, accompanied by significant increases in testosterone and reductions in estradiol. These findings are consistent with previous work by El Bardisi et al. [21] and Wood et al. [24] and suggest that functional sperm parameters may be more responsive to metabolic recovery than sperm concentration.

Procedure-specific differences were observed. Participants undergoing OAGB or RYGB experienced greater reductions in estradiol and larger gains in motility compared with that receiving sleeve gastrectomy. These findings align with current international guidelines, which recognize the enhanced metabolic benefits of bypass procedures in selected patients. The endocrine improvements observed in our study support the growing recognition of MBS as a therapeutic option not only for weight management but also for correcting obesity-related reproductive dysfunctions—an area of increasing interest in BMC Surgery due to its focus on metabolic mechanisms.

Greater improvements were observed in patients undergoing OAGB or RYGB; however, these findings should be interpreted cautiously due to the exploratory nature of the analysis and limited subgroup sizes.

Transient postoperative declines in semen quality occurred in a small subset of participants and were associated with micronutrient deficiencies. This underscores the need for rigorous postoperative nutritional monitoring, particularly during rapid weight-loss phases.

Clinically, these results highlight the importance of incorporating reproductive counseling into bariatric evaluation pathways. For reproductive urologists, the findings suggest that MBS may be considered as part of a broader management plan for men with obesity-related infertility.

Future research should include controlled comparative designs, integrate lifestyle-based interventions as comparators, and assess fertility outcomes such as pregnancy and live birth rates.

These findings may assist bariatric and reproductive clinicians in counseling obese men seeking fertility improvement.

The absence of separate free testosterone reporting represents a limitation and should be considered when interpreting androgen-related outcomes.

These limitations highlight the exploratory nature of procedure-specific comparisons and underscore the need for larger, controlled studies focusing on reproductive outcomes after bariatric surgery.

The subgroup analyses comparing different bariatric procedures were exploratory and limited by small sample sizes.

## Conclusion

Metabolic and bariatric surgery was associated with significant improvements in semen motility, vitality, and hormonal balance among infertile men with obesity. Although greater improvements were observed following OAGB and RYGB compared with sleeve gastrectomy, these findings should be interpreted cautiously due to the exploratory nature of the subgroup analyses and the limited sample size. These findings suggest that bariatric surgery may represent a potential therapeutic strategy for obese men with infertility and hypogonadism, although larger controlled studies are required to confirm these effects.

### Limitations

This study has several limitations. The sample size was relatively small and derived from a single center, which may limit generalizability. The absence of a non-surgical control group prevents definitive causal inference regarding the effect of bariatric surgery on reproductive parameters. Subgroup comparisons between surgical procedures were exploratory due to limited numbers in each group. Additionally, sperm DNA fragmentation was not assessed, free testosterone was not reported as a separate endpoint, and no pregnancy or live birth outcomes were recorded during the follow-up period. Future multicenter studies with larger cohorts and longer follow-up are needed.

## Data Availability

The datasets generated and analyzed during the current study are available from the corresponding author on reasonable request.

## Abbreviations

MBS: Metabolic and bariatric surgery
SHBG: Sex hormone-binding globulin
TT: Total testosterone
FT: Free testosterone
PPIs: Proton pump inhibitors
AZF: Azoospermia Factor
RYGB: A Roux-en-Y gastric bypass
SG: Sleeve gastrectomy (SG)
OAGB: One anastomosis gastric bypass
ICSI: Intra-cytoplasmic sperm injection
OAT: Oligoasthenoteratozoospermia
